# Reviews

**Published:** 1879-12

**Authors:** 


### REVIEWS.

---

**Atlas of Skin Diseases.** By LOUIS A. DUHRING, M. D., Professor of Skin Diseases in the Hospital of the University of Pennsylvania, &c. Philadelphia: J. B. Lippincott & Co. 1879. Part vi.

This number contains three elegant chromo-lithographic plates, illustrating with great accuracy typical cases of syphiloderma (pustulosa), erythema nodosum and seborrhœa. The author, taking each case as a text, gives a very clear description of the disease, its pathology, diagnosis and treatment. He then presents almost a clinical lecture upon each disease, the plate serving in place of the patient. We regard this method of treating this otherwise difficult class of diseases as meeting, more completely than any other with which we are familiar, the wants of the profession. We commend the work, and earnestly hope it will meet with the favor it justly merits. L.

**Lecture Notes on Physiology and Pathology.** By VICTOR C. VAUGHAN, M. D., Ph. D., Lecturer on Medical Chemistry in the University of Michigan. Second Edition, Revised and Enlarged. Ann Arbor Printing and Publishing Co , Ann Arbor, Michigan.

We are much pleased with this enlarged edition of Dr. Vaughan's "Lecture Notes." The book is an excellent one: clear, concise, convenient and practical. It gives the latest accepted facts of physiological science, as far as they relate to the subject in hand, without entering into the conflicting theories and statements which have been held concerning the phenomena exhibited by animal bodies. A large part of it is devoted to the study of the urine in health and disease. The value of this part of the work will be greatly enhanced by a book of plates, now in press, representing the crystals of the most important of the substances here studied. When these charts appear we will again call the attention of our readers to this excellent work.

D.

---

**Physiology and Histology of the Cerebral Convolutions; also, Poison of the Intellect.** By CHAS. RICHET, M. A., M. D. Translated by EDWARD P. FOWLER, M. D New York: Wm. Wood & Co.

The tendency of recent investigation is to show that mental phenomena are the result of physical conditions. The author of this monograph follows out the same line of study, to determine, if possible, the function of the gray matter in the convolutions as affecting sensation and motion. After describing the anatomy of this part of the brain, the results of vivisection are narrated at length, and compared with those obtained by other experimenters. The writer is careful not to state any conclusions in a dogmatic manner, but the data are fairly presented to the reader, and he is left, to a great extent, to draw his own inferences. It is evident that much remains to be learned concerning every branch of mental physiology, but the facts we find recorded here, and in fact, whatever positive knowledge we have on the subject, must lead us to consider thought simply as "the physiological function of the brain."

H.

**Obstetrical Operations.** By ROBERT BARNES, M. D., F R. C. P. New York: D. Appleton & Co.

This work of about 600 pages octavo, with over one hundred illustrative engravings, is one of rare value. We have read it with care, and in so doing made numerous notes with a view to a more or less critical review. The notes are, however, so numerous and the points of interest so abundant that we can only advise our readers, who have not already read it, to immediately obtain the work. The articles are original and practical, and the illustrations, the author tells us, were in many instances drawn at the bedside. We agree with Pagot, when in his preface to the French edition, he says, "I am happy to be able to bear witness to the numerous clinical truths sown over every page of this book. . . The description of the instruments, the application of the forceps, cephalotripsy, embryotomy, cæsarian section, the practical reflections on narrowing and malformation of the pelvis, ruptures of the uterus, placenta prævia, hemorrhage, and in fact, all the great questions in obstetrics, are treated with accuracy and good sense. At each instant, by some remark or other, is revealed a superior mind, ripened by having seen much and meditated much."

The suggestions and instructions in many instances deviate from the accustomed path, but we have failed to find any which, upon reflection, did not present themselves as worthy of acceptance. Once more we say get the book, and read and study it for yourselves. V. P.

---

**Diseases of the Throat and Nasal Passages.** A Guide to the Diagnosis and Treatment of Affections of the Pharynx, Œsophagus, Trachea, Larynx, and Nares. By J. SOLIS COHEN, M. D., Lecturer on Laryngoscopy, in Jefferson Medical College, etc., etc. New York: William Wood & Co.

This is the second edition of the author's well-known work, and the anticipations with which the profession have looked forward to its appearance have been more than fully realized. We consider it an honor for American medical literature to possess a work like this, by all means the largest, most perfect and most complete handbook on diseases of the upper air-passages yet published. The second edition is so much enlarged and amended that it may be considered a new work. Among other things, the author's valuable lectures on the Surgery of the Larynx, Nares and Trachea, on Fetid Coryza, Sore Throat and Diphtheria are included in this edition. The fifth chapter, on Diphtheria, is especially interesting. The author is an ardent advocate of tracheotomy, "if there be such evidence of false membranes in the larynx and trachea as to threaten suffocation. There is no insuperable contraindication, unless it be the evidence of the deposit of fibrine in the cavities of the heart. It is a chance for life which should not be denied the patient—and the sooner the operation is performed after it is indicated, the better the hope of success." It is the same opinion advocated by the great European surgeons, led by Trouseau.

The illustrations are numerous and excellent, and the book as a whole is a work which no physician, who has any desire to follow the progress of medical science, can afford to be without.

M.

---

**Students' Pocket Medical Lexicon**: Giving the Correct Pronunciation and Definition of all Words and Terms in general use in Medicine and the Collateral Sciences, etc., etc. By ELIAS LONGLEY. Philadelphia: Lindsay & Blakiston. 1879. 303 pp.

The author's preface to this little work takes up four closely printed pages, in which he aims to establish the need of a new lexicon "to enable students, and even old practitioners, to understand all they hear and read on professional subjects." The English mode of pronunciation is adopted in preference to the Continental, to which also is added the phonetic orthography of each word. Inasmuch as many students have not been favored with a liberal education, the system, which this work adopts, has many advantages deserving of special commendation. We regard this lexicon a valuable aid to the student for whom it is designed.

L.

**First Step in Chemical Principles.** An Introduction to Modern Chemistry, intended especially for Beginners. By HENRY LEFFMANN, M. D., Lecturer on Toxicology in the Summer-school of Jefferson Med. College. Philadelphia: Edward Stevens & Co. 1879. 50c.

To those commencing the study of chemistry and to medical students, we would particularly recommend this little book. The mysteries of equivalents, atomicities and chemical notation, which beginners find it so hard to understand, are here plainly and simply explained, as only one familiar with the difficulties which the students encounter could do it. This little book should be in the hands of every medical student. D.

---

**Winter and its Dangers.** BY HAMILTON OSGOOD, M. D. Philadelphia: Lindsay & Blakiston. 1879.

This is number six of the American Health Primer Series, and is devoted to the consideration of dangers arising from errors in dress; carelessness and ignorance in bathing; inattention to pulmonary food; dangers from overheated air; indifference to sunshine; sedentary life and neglect of exercise; dangers of school-life in winter; winter amusements, etc., etc.

These subjects are treated in a plain and practical manner, suited to the non-professional reader. The work is an exceedingly useful one, and contains information in a condensed and convenient form, suited for general distribution. L.

---

**The Multum in Parvo Reference and Dose Book.** By C. HENRI LEONARD, M. A., M. D. 3d Edition, revised and enlarged. Detroit.

This new edition of Leonard's Dose Book contains the doses of over 2500 preparations. We find in it many of the "out of the way" remedies, of which as being little used, the practitioner is very apt to need a reminder as to dose, etc. Some 225 *new* preparations have also been added to the list, and also tables giving the relation of metric weights, etc; a list of incompatible and other information, which makes the little book indeed a "multum in parvo." D.

**Photographic Illustrations of Skin Diseases.** By GEORGE HENRY FOX, M. D., Professor of Dermatology in Starling Medical College. New York: E. B. Treat, 805 Broadway.

We have received the first part of this elegant work, which will be completed in twelve parts, containing in all forty-eight colored photographs taken from life. The first part contains four plates, Comedo, Acne Vulgaris, Lepra Tuberosa, Elephantiasis, each accompanied with a short but distinct and clearly-written text, in which the rules for treatment are laid down. The plates are a marvel of successful representation of skin diseases, and could scarcely be surpassed. Any physician will learn more about the diagnosis of these diseases by the aid of this work, in an hour, than by reading a large book; they are indispensable for the teacher and the student, as much as for the practicing physician. They are recommended highly by the leading dermatologists of this country. We predict a great success for the work. Considering the excellence of the plates, the price, $2 for each part, is moderate. M.

---

**Posological Tables**; including all the Officinal and the Most Frequently Employed Unofficinal Preparations. By CHARLES RICE, Chemist, Department of Public Charities and Correction, &c. Revised and Approved by Members of the Medical Boards of Bellevue and Charity Hospitals. New York: William Wood & Co.

This is a very well-arranged and complete dose list, and will be found very convenient to the physician as a book for ready reference. A table of equivalents of weights and measures of the decimal and common system is appended, also rules for their ready conversion. D.

